# Variations in Total Protein and Amino Acids in the Sequenced Sorghum Mutant Library

**DOI:** 10.3390/plants12081662

**Published:** 2023-04-15

**Authors:** Adil Khan, Nasir Ali Khan, Scott R. Bean, Junping Chen, Zhanguo Xin, Yinping Jiao

**Affiliations:** 1 Institute of Genomics for Crop Abiotic Stress Tolerance (IGCAST), Department of Plant and Soil Science, Texas Tech University, Lubbock, TX 79409, USA; 2 Grain Quality and Structure Research Unit, Center for Grain and Animal Health Research, USDA-ARS, 1515 College Ave., Manhattan, KS 66502, USA; 3 Plant Stress and Germplasm Development Unit, Crop Systems Research Laboratory, USDA-ARS, 3810, 4th Street, Lubbock, TX 79424, USA

**Keywords:** *Sorghum bicolor*, kafirins, ethyl methanesulfonate (EMS), amino acids

## Abstract

Sorghum (*Sorghum bicolor*) is the fifth most important cereal crop worldwide; however, its utilization in food products can be limited due to reduced nutritional quality related to amino acid composition and protein digestibility in cooked products. Low essential amino acid levels and digestibility are influenced by the composition of the sorghum seed storage proteins, kafirins. In this study, we report a core collection of 206 sorghum mutant lines with altered seed storage proteins. Wet lab chemistry analysis was conducted to evaluate the total protein content and 23 amino acids, including 19 protein-bound and 4 non-protein amino acids. We identified mutant lines with diverse compositions of essential and non-essential amino acids. The highest total protein content in these lines was almost double that of the wild-type (BTx623). The mutants identified in this study can be used as a genetic resource to improve the sorghum grain quality and determine the molecular mechanisms underlying the biosynthesis of storage protein and starch in sorghum seeds.

## 1. Introduction

Sorghum (*Sorghum bicolor*), belonging to the *Poaceae* family, is the fifth major cereal crop worldwide, after wheat, rice, corn, and barley, in terms of grain productivity [1]. Sorghum is widely used in animal feed, fodder, and high-value products, such as syrup and bioethanol, making it a promising candidate for multipurpose feedstock [2,3]. Because of its excellent resistance to drought in agro-ecological zones with limited rainfall, where the yield of other cereal crops is insufficient, sorghum is considered as a potential alternative to nutrient-enriched food [4]. Sorghum is a major crop in the semiarid and arid areas of the world, particularly in Africa, where it is a staple meal for a sizable portion of the population [5]. However, it has lower nutritional value compared to other crops [6,7]. Therefore, it is important to develop improved sorghum varieties with higher nutritional value.

Sorghum grain can have a wide range of protein content, but on average contains approximately 11% crude protein [8,9]. The most prevalent proteins, prolamins (kafirins in sorghum), are present in the endosperm with the majority found in spherical protein bodies [10]. Notably, kafirin accounts for approximately 48–70% of the total protein content in whole grains and up to 80% of the protein content in decorticated kernels [2,11]. Starch is the main carbohydrate in sorghum grains. Similar to protein content, total starch can vary widely but on average the starch content in sorghum is ~70%. Starch consists of two polysaccharides: amylopectin, accounting for 70–80% of the starch, and amylose constituting the remaining 20–30% [10,12]. Similar to maize and other cereal grains, sorghum grains have low quantities of oil and other lipids, generally 2–4% on a weight basis [13].

Sorghum storage proteins exhibit a strong sequence similarity to the maize storage proteins, zeins [14]; however, sorghum proteins are more hydrophobic and have a greater number of cross-linked fractions than the maize proteins, indicating their greater propensity to create intermolecular disulfide cross-connections and protein aggregates [14,15]. The combination of protein bodies and matrix protein in the endosperm forms a protein barrier around the starch granules, which in turn inhibits amylolytic enzymes from hydrolyzing native and processed starch [16,17]. Starch-gelatinizing is also negatively impacted by protein structures (protein bodies and matrix protein) [3,15].

All cereal grains are low in lysine and developing germplasm with high lysine content may result in significant trade-offs, such as poor grain quality, with a soft and floury endosperm texture, in cereals [18]. Modifier genes increasing zein accumulation have been reported to dissociate the higher lysine content in the floury endosperm in maize [19,20]. For example, the opaque-2 mutant is a potential candidate for enhancing the maize protein quality by increasing its lysine and tryptophan levels [19]. Interestingly, sorghum possesses a higher amount of the polymerization of its prolamins (kafirins) [21,22]. Polymerization of kafirins results in reduced protein digestibility [23]. In previous studies, chemical mutagenesis (diethyl sulfate treatment) was used to produce the highly digestible high-lysine (dahl) mutant *P721Q* (Q for opaque) from *P721N* (N for normal) in sorghum [21,24,25]; the mutant produced floury kernels and non-vitreous phenotypes, including lysine, because of the lack of accumulation of kairine [24].

Depending on the ability of the human body to synthesize amino acids, amino acids can be divided into essential and non-essential types [26,27,28]. Essential amino acids cannot be synthesized by the human body and need to be provided through external resources, whereas non-essential amino acids can be synthesized by the human body and are not required from external sources [9,29]. People in low-income nations with subsistence level agricultural systems who depend on a few specific crops with an imbalanced amino acid composition have more health issues caused by the inadequate intake of crucial amino acids [9]. Addition of chemically generated amino acids to manufactured animal and human food is not cost-effective in low-income countries. Therefore, it is necessary to increase the concentration of essential amino acids and protein content in food crops to aid in crop development. Various conventional and transgenic techniques have been successfully used to enhance the protein content and amino acid balance in plant seeds. Lysine-rich sorghum germplasm was created via transgenesis of the gene *HT12* to improve the nutritional quality of the sorghum grain [30,31,32,33]. However, these techniques require more time and sophisticated lab facilities, which are not available in low-income countries, necessitating the development of an eco-friendly and economical approach that provides immediate agronomic benefits to the farmers. 

The mutant populations in plants have been widely used in plants as a resource of genetic diversity for crop functional genomics and breeding applications. It has been demonstrated that mutant populations are powerful resources for gene function study [34]. Large-scale plant mutant populations could be produced in several ways, including T-DNA insertion and physically- or chemically-induced mutagenesis [35]. T-DNA-based insertional mutagenesis is impractical to generate large mutant populations for crops such as sorghum, due to the lack of a highly efficient transformation system [36]. Therefore, chemical mutagens such as ethyl methanesulfonate (EMS) are often employed for seed mutation because they are efficient to create high frequency point mutations [37]. The EMS chemical could induce high density stable point mutations at the genome level. The desirable mutants may be used directly in breeding without raising any concerns about genetic modification [37]. EMS-induced mutagenesis has been utilized effectively in Arabidopsis [38,39,40], rice [41,42], wheat [43,44], maize [45,46], barley [47], sorghum [48,49], and tomato [50]. For example, it has been shown that EMS-induced mutagenesis is a successful method for obtaining genetic resources for high-quality rapeseed breeding, particularly for the creation of cultivars with high oleic acid and low linolenic acid in Brassica napus [51,52]. Likewise, Soybean mutagenesis has been widely used in characterized loci controlling important agronomic traits such as seed quality. For example, the FAD2-1A gene (Glyma.03G144500) has been shown to influence the concertation of linoleic acid. Similarly, mutations in the RS2 (raffinose synthase; Glyma.03G137900) genes result in higher sucrose levels and lower concentrations of raffinose and stachyose oligosaccharide [53,54,55]. Similarly, another EMS-based mutant population was used to investigate the mechanism of oil production and generate germplasms for Brassica napus breeding [56]. In maize, the Waxy1 line was created by EMS-based mutagenesis, which could produce the low expression level of granule-bound starch synthase I and lead to the low level of amylose but high level of amylopectin in seed [57,58]. OsNF-YB1 knockout in rice led to altered rice quality due to the changes in grain size, amylose, total starch, crude fiber, and lipid content, as well as increased protein content [59]. In recent years, induced mutations have been extensively used for breeding annual oilseed crops [60,61,62,63]. The maize Opaque-2 (O2) mutant has been used in breeding to produce new maize verities with high lysine content and reduced zein to glutenin ratio [64,65]. The maize O2 mutant has been used in breeding to produce new maize verities with high lysine content and reduced zein to glutenin ratio [64,65]. Hence, the mutant germplasm produced by EMS that has the potential to improve seed quality and other agronomic performance may be employed as a complementing technique in the genetic improvement of cereal crops.

Although several EMS mutant populations have been developed previously, none of the sorghum mutant populations have been screened extensively for seed quality traits [48,49,66,67,68]. Sorghum has been underutilized in grain quality studies due to the limited availability of genetic resources. To facilitate the functional genomics research of sorghum, we previously established a large sorghum mutant population containing more than 6400 M2 pools [48,49]. More than 1.8 million canonical EMS-induced mutations were discovered from the whole-genome sequencing of 256 mutant lines, which covered more than 95% of the sorghum genome. Interestingly, 97.5% of the generated mutations were not discovered in the natural variants. This population has been of great value in characterizing important traits in sorghum, including epicuticular wax, seed size, and inflorescence development [49,69,70]. This mutant population has sufficient mutation density and low cross-fertilization, making it a useful genetic resource for examining the functions of sorghum genes. The underlying mutant resources, coupled with compressive grain quality analysis for essential and non-essential amino acids provide an efficient platform for functional validation of genes related to important seed traits in sorghum.

In this study, we analyzed 23 amino acids in 206 ethyl methanesulfonate (EMS)-based sorghum mutant lines to provide a new germplasm resource for sorghum grain quality improvement. EMS mutagenesis technique has gained momentum for generating mutant populations due to its high frequency of point mutations [71,72]. Herein, we used a wet chemistry approach to determine the levels of essential and non-essential amino acids in EMS-based mutant and wild-type sorghum crops to compare their nutrient dynamics. Our study may serve as a robust genetic resource for enhancing the sorghum genetic potential to improve its grain nutrient content and aid in the development of effective sorghum breeding strategies.

## 2. Results

### 2.1. Variations in the Seed Amino Acid and Total Protein Levels in the Mutagenized Sorghum Population

**Figure 1:**
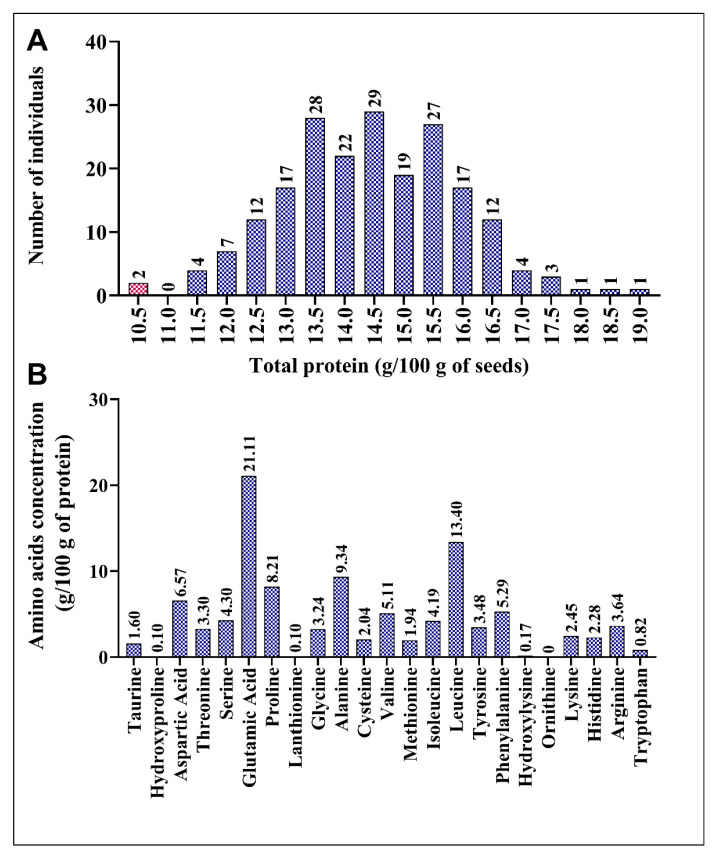
Variations in the total protein content in the mutant population and amino acid distribution in BTx623. (**A**) Variation in the total protein content and number of mutant lines. (**B**) Composition of amino acids in the total protein of BTx623. Control line (BTx623) is indicated by a red bar in each panel.

To identify any variations in amino acid levels in the sorghum mutant population, we analyzed the M3 generation of 206 mutant lines. This mutant population was generated via EMS treatment of the reference genome line, BTx623, through single-seed descent. Each mutant line contained, on average, 7660 mutations. The overall mutation density in the population was 11 SNPs/Mb, with a range varying from 0.02 to 22.5 SNPs/Mb [49]. We conducted wet lab chemistry analysis to determine the seed protein composition, including total protein content and concentration of various amino acids in the mutant population. Wet lab chemistry analysis provides accurate and precise measurements of the content of each amino acid [73,74]. Total protein content and distribution of 23 amino acids, including 19 protein-bound and 4 non-protein amino acids, are depicted in Figure 1A. Total protein content (10.28–19.01%) in 206 accessions indicated new genetic variability due to induced mutagenesis (Figure 1A). Surprisingly, the total protein content was higher in all mutant lines than in the BTx623 line (10.35%). ARS197 (19.14%), ARS152 (18.45%), and ARS (18.04%) showed increases in total protein content of 84.92, 78.26, and 74.29%, respectively (Figure 1A). In wild-type BTx623, the most abundant amino acid in sorghum seeds was glutamic acid (21.11%), followed by leucine (13.40%), alanine (9.34%), and proline (8.21%) (Figure 1B). The minimum and maximum concentrations of each amino acid in the mutant population are listed in Table 1. Major amino acids, hydroxylysine, methionine, taurine, cysteine, tyrosine, tryptophan, hydroxyproline, lanthionine, and ornithine, exhibited > 20% difference between their minimum and maximum concentrations, indicating diversity in the mutagenized population (Table 1).

**Figure 2:**
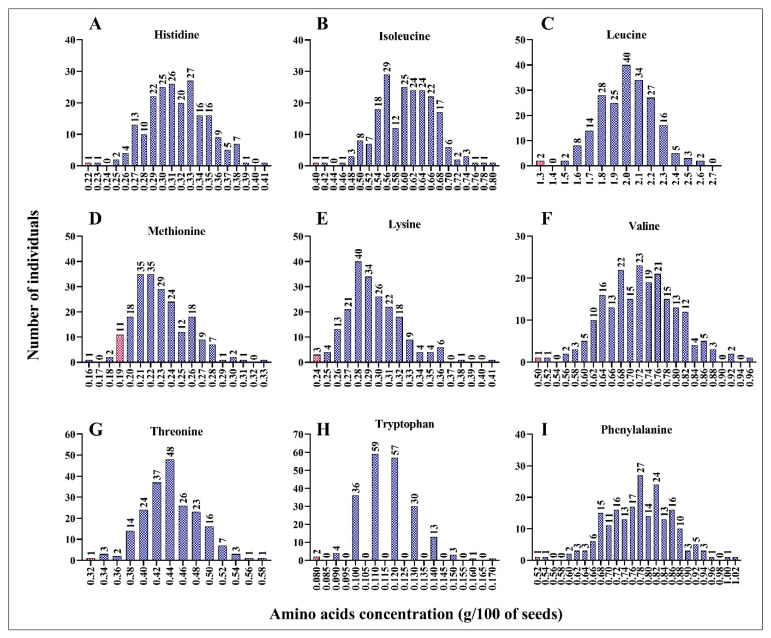
Mutant lines with various concentrations of the essential amino acids, histidine (**A**), isoleucine (**B**), leucine (**C**), methionine (**D**), lysine (**E**), valine (**F**), threonine (**G**), tryptophan (**H**), and phenylalanine (**I**). The x value indicates g/100 g of dry seed sample. Total protein was extracted from 100 g of seeds and the amino acids were quantified. Control line (BTx623) is indicated by a red bar in each panel.

**Figure 3:**
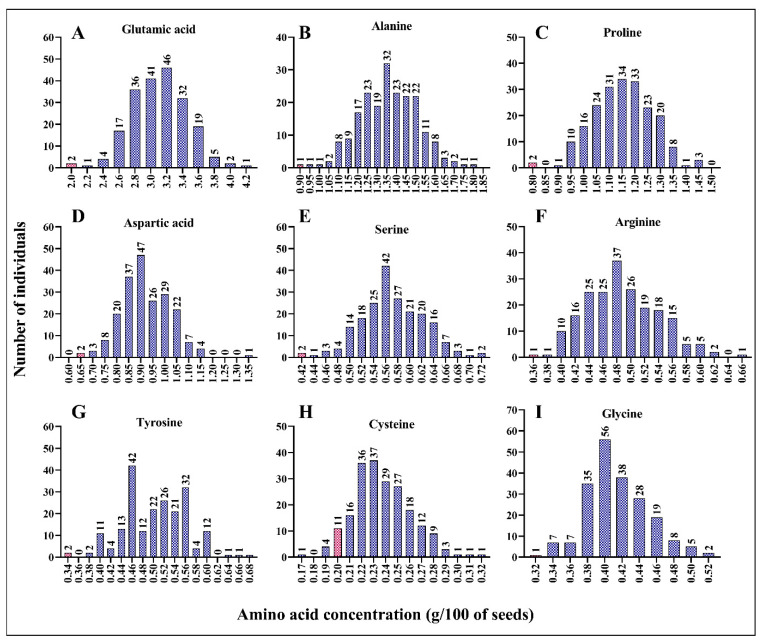
Mutant lines with various concentrations of the non-essential amino acids, glutamic acid (**A**), alanine (**B**), proline (**C**), aspartic acid (**D**), serine (**E**), arginine (**F**), tyrosine (**G**), cysteine (**H**), and glycine (**I**). The x value indicates g/100 g of dry seed sample. Total protein was extracted from 100 g of seeds and the amino acids were quantified. Control line (BTx623) is indicated by a red bar in each panel.

Using the wet lab chemistry approach, we measured the concentrations of essential (histidine, isoleucine, leucine, lysine, methionine, phenylalanine, threonine, tryptophan, and valine) and non-essential (alanine, arginine, asparagine, aspartic acid, cysteine, glutamic acid, glutamine, glycine, proline, serine, and tyrosine) amino acids in each mutant line. We found a positive correlation between the concentration of each amino acid and the total protein content (Figure S1). As shown in Figure S1, the concentration of each amino acid increased with the increase in total protein content in the seeds. Compared to leucine, methionine, tryptophane, and phenylalanine (R^2^ = 0.594, 0.485, 0.492, and 0.632, respectively), histidine, lysine, threonine, and valine (R^2^ = 0.671, 0.918, 0.746, and 0.720, respectively) exhibited stronger correlations (Figure S2). This result suggests that the mutant lines with a high percentage of total protein had high concentrations of essential amino acids. Compared to the wild-type line, ARS158, ARS125, and ARS197 mutant lines had double the concentrations of important amino acids. Histidine concentration was 0.41 g/100 of dry seed in ARS158 and ARS125 mutant lines compared to 0.22 g/100 of dry seed in BTx623 (Figure 2A). Similarly, isoleucine, leucine, lysine, and phenylalanine concentrations were doubled in the ARS197 mutant line compared to that in the wild-type line. ARS197 had high concentrations of isoleucine, leucine, lysine, and phenylalanine, indicating positive correlations among these amino acids (Figure 2A–I). Compared to BTx623, the mutant lines had higher concentrations of the essential amino acids, histidine, isoleucine, leucine, lysine, and phenylalanine. However, ARS159, showed lower concertation of methionine (0.16 g/100 g of dry seed) than BTx623 (0.19 g/100 g of dry seed). Concentrations of non-essential amino acids showed a similar trend in the mutant population (Figure 3A–I). Similar to essential amino acids, non-essential amino acids also showed a positive correlation with the total protein content (Figure S2). All mutant lines had higher concentrations of alanine, proline, serine, arginine, and tyrosine than BTx623. However, five mutant lines exhibited lower concentrations of cysteine than BTx623 (Figure 3A). A similar trend was observed for the non-protein amino acids, ornithine, taurine, and hydroxyproline (Figure S2).

### 2.2. Variability in the Kernel Structure, Composition, and Starch Content

**Figure 4:**
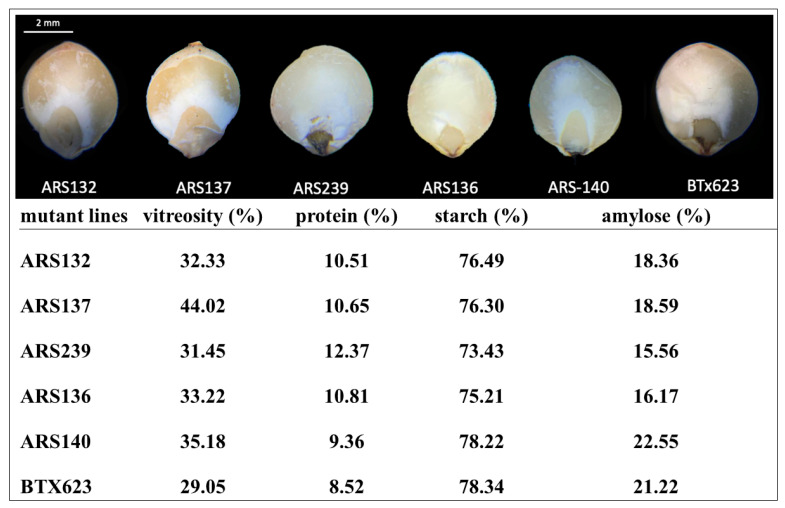
Variability in the kernel structure, composition, and starch content in five ethyl methanesulfonate (EMS) mutant sorghum lines and controls (BTx623).

To further investigate the effects of mutations on the kernel structure composition and starch content, seeds from five mutant lines (ARS132, ARS137, ARS239, ARS136, and ARS140) and BTx623 were analyzed using near-infrared (NIR) spectroscopy. These mutant lines were randomly selected for comparison with BTx623. As illustrated in Figure 4, the mutations in these five lines affected the proportion of floury and vitreous endosperm, vitreosity, and the seed protein, total starch, and amylose contents. The proportion of vitreous endosperm was higher than that of floury endosperm in ARS132, ARS137, ARS136, and ARS140 lines (Figure 4). However, ARS239 showed a pattern similar to that of BTx623. Floury and vitreous endosperm traits are directly related to the starch digestibility rate. These components are unique in their composition and their relative proportions influence the final chemistry of the grain. In addition, the grain composition is closely intertwined with the physical structure [75]. Hence, the mutant lines generated in this study may be a useful resource for investigating the effects of floury and vitreous endosperms on the starch digestibility rate and other traits of sorghum seeds. ARS132, ARS137, ARS136, and ARS140 mutant lines had higher vitreosity than BTx623 and ARS239 lines (Figure 4), indicating that the rate of vitreosity is directly correlated with the proportion of floury and vitreous endosperm. We also quantified the total protein content via NIR spectroscopy and found a correlation of 0.857 with wet lab chemistry analysis, indicating the high quality and reliability of the data generated in this study. ARS239 had a higher protein content than the other mutant lines and BTx623, suggesting that the proportion of vitreous endosperm is negatively correlated with the total protein content. Compared with BTx623, no obvious changes were observed in any of the mutant lines. Interestingly, amylose content decreased by 26.673% in ARS239 compared to that in BTx623, suggesting that the floury endosperm has more amylose content than the vitreous endosperm (Figure 3). These results indicate that the mutant lines generated in this study have sufficient variability and can be used as a valuable resource for functional genomics research and sorghum breeding in the future.

### 2.3. Correlation between the Total Protein and Amino Acid Levels in the Mutagenized Sorghum Population

**Figure 5:**
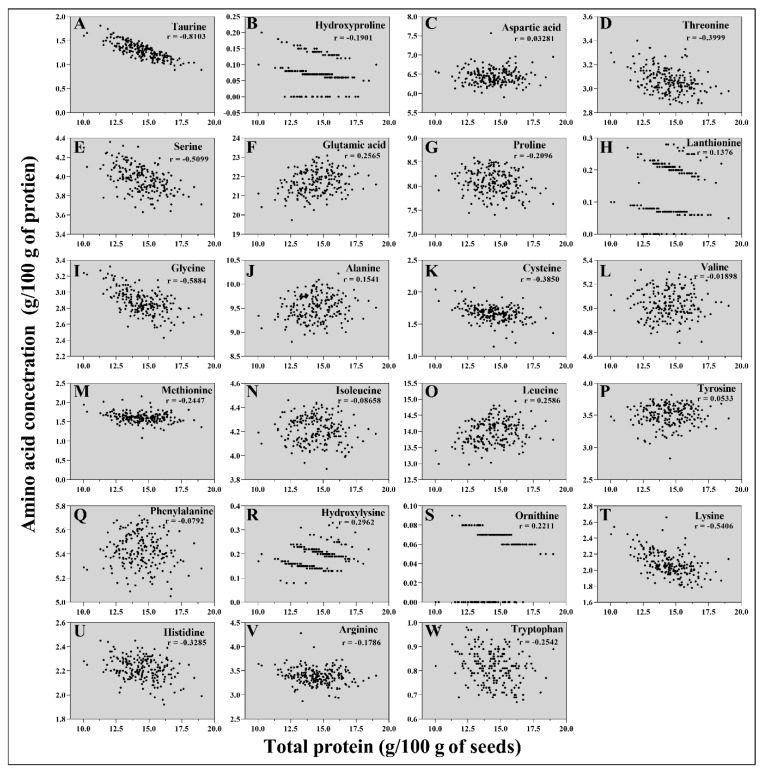
Correlations between the concentrations of amino acids (%), taurine (**A**), hydroxyproline (**B**), aspartic acid (**C**), threonine (**D**), serine (**E**), glutamic acid (**F**), proline (**G**), lanthionine (**H**), glycine (**I**), alanine (**J**), cysteine (**K**), valine (**L**), methionine (**M**), isoleucine (**N**), leucine (**O**), tyrosine (**P**), phenylalanine (**Q**), hydroxylysine (**R**), ornithine (**S**), lysine (**T**), histidine (**U**), arginine (**V**), and tryptophan (**W**), and the total protein concentration (g/100 g of seeds) in the seeds of mutant lines. Each point indicates the amino acid concentration in each mutant line. The r value represents the Pearson correlation.

To determine the correlations between each amino acid concentration and the total protein content, the concentrations of each amino acid (g amino acid/100 g protein) relative to the total protein content were calculated. Glutamic acid, lanthionine, alanine, leucine, hydroxylysine, and ornithine showed positive correlations with the total protein content (Figure 5), suggesting that their concentrations increase relative to the total protein content. Notably, glutamic acid, alanine, and leucine were the three most abundant amino acids in wild-type seeds. Taurine, hydroxyproline, threonine, serine, proline, glycine, cysteine, methionine, lysine, histidine, arginine, and tryptophan showed negative correlations with the total protein content. For example, the total protein content was increased by 89.34% in the ARS197 line compared to that in BTx623, but the lysin content was decreased (2.16 g/100 g of protein) in the ARS197 line compared to that in BTx623 (2.39 g/100 g of protein). de Borja Reis, et al. [76] reported similar findings in soybeans. Aspartic acid, phenylalanine, isoleucine, valine, and tyrosine were not significantly related to the total protein concentration in the mutagenized sorghum population (Figure 5). These results indicate that the amino acid to protein ratio directly affects the nutritional value of sorghum seeds and should be specifically considered in the development of high-protein varieties. 

## 3. Discussion

Sorghum is the fifth most important cereal crop that is grown as a food and feed crop worldwide. In comparison to wheat, rice, and maize, sorghum may have lower nutritional value, which is directly affected by the protein quality of the grain. Along with lower protein digestibility, sorghum is low in essential amino acids, especially lysine. Therefore, it is necessary to develop new germplasm sources, such as mutant populations, with variability in seed composition, particularly in essential amino acid content. In this study, mutant lines were generated using an EMS-based approach. These mutant lines showed variation in seed amino acid concentrations compared to those in the BTx623 line and may be used in future sorghum breeding programs.

In this study, the mutants exhibited wide variability in the total protein content and 23 amino acids, including 19 protein-bound and 4 non-protein amino acids. Lasztity [77] and De Mesa-Stonestreet et al. [78] revealed that the total protein content in elite sorghum lines ranged from 6 to 18%, with an average of 11%. The lines generated in this study showed 10–19.2% total protein content (Figure 1A), which varied significantly in the mutant lines. Compared to BTx623, all mutant lines showed a higher total protein content (Figure 1A). Similar results have been reported in a soybean EMS-based mutant population [79]. In addition, the essential amino acid content was positively correlated with the total protein content. Several mutant lines with altered amino acid content were also observed. Therefore, the mutant population developed in this study can be used as a resource to enhance the sorghum seed quality by increasing its essential amino acid content.

Our mutant lines can aid in the identification of genes affecting the seed quality. We identified promising mutants with increased total protein and essential amino acid contents in this study. We also observed positive correlations among various amino acids in the ARS197 mutant line. Hence, this mutant line can be used to determine the correlations among various amino acids. Mutants showing variability in kernel weight, kernel size, amylose content, virtuosity, and starch content may also exhibit a high yield. Therefore, the mutant lines generated in this study can be used in sorghum breeding programs to improve the seed quality, such as increase the essential amino acid content and digestibility, and other important traits, such as yield, kernel hardness, and starch content.

## 4. Material and Methods

### 4.1. Generating a Mutant Population

We generated a mutant population via EMS treatment, as previously described [49]. Briefly, approximately 100 g of BTx623 seeds was treated with 0.1–0.3% EMS (*v*/*v*) for 16 h at 50 rpm on a rotary shaker. Seeds were thoroughly washed for 5 h in tap water at an ambient temperature. During incubation, the water was repeatedly changed every 30 min. Seeds were air-dried and planted in the field at a density of 120,000 seeds per hectare. To prevent cross-pollination, the panicles of each plant were covered with a 400-weight rainproof paper pollination bag before anthesis. Mature seeds were harvested from each plant and advanced to M2 generation by growing the resulting seeds one row per head, and the panicle was bagged before anthesis (three individuals in each row). One panicle progressed to M3 generation. Ten panicles were bagged for each M3 head row and pooled as M4 seeds, which were distributed to the end users upon request. Sample preparation, DNA extraction, variation detection and function, and prediction characterization of *Sorghum* EMS-induced single-nucleotide polymorphisms have been previously described [49]. For the current study, mutant collections of 256 lines were planted at the USDA-ARS Plant Stress and Germplasm Development Research Unit, Lubbock, Texas (latitude 33°35′ N, longitude 101°53′ W, and altitude 958 m) in 2017. The soil type is an Amarillo fine sandy loam (fine-loamy, mixed, superactive thermic Aridic Paleustalfs). Before planting, a mixture of bulk ammonium sulfate and mono ammonium phosphate was applied to the field, calculated to achieve levels of 65 kg nitrogen and 27 kg phosphorous per hectare. An augmented design with no replicates was used. The plot size is four rows of 4.67 m long with 1.02-m row spacing. Sorghum seeds were planted at 80 per row at a depth of 3 cm using a John Deere MaxEmerge Planter. The irrigated plots received 5 mm of water per day from underground drip lines located on 1.02-m centers as needed. Fifty grams of dry seed from 206 lines were provided to Agricultural Experiment Station Chemical Laboratories of the University of Missouri-Columbia for total protein and composition of amino acids. The remaining 50 lines with low seeds yield were not included.

### 4.2. Total Protein and Amino Acid Extraction and Analysis

Total protein and amino acid contents were analyzed by the Agricultural Experiment Station Chemical Laboratories of the University of Missouri-Columbia (https://aescl.missouri.edu/index.html, accessed on 1 January 2023) following the standard methods of the Association of Official Analytical Chemists (AOAC). Briefly, crude seed protein content was calculated from the total nitrogen content using the Kjeldahl method [80]. The complete amino acid profile was analyzed via cation-exchange chromatography coupled with post-column ninhydrin derivatization and quantitation according to the AOAC Official Method 982 [81].

### 4.3. NIR Analysis of the Five Sorghum Mutant Lines

Grain composition of five selected sorghum mutant lines was determined using NIR, as previously described [82]. The vitreosity of the samples was determined via image analysis of the cut sorghum kernels, as described in [83]. 

## 5. Conclusions

The sorghum mutant population showed significant variability in total protein content, along with variability in 19 protein-bound and 4 non-protein amino acids. Mutants with doubled amounts of total protein and essential amino acids, such as histidine, leucine, and lysine, have great potential for improving sorghum seed quality. In addition, we observed variability in kernel weight, kernel size, amylose content, virtuosity, and starch, which could also be used to improve important traits of sorghum. The mutant lines that exhibited interesting variability in seed composition generated in the current study will be freely available for sorghum breeding programs.

## Figures and Tables

**Table 1:** Summary of the amino acid analysis of 206 sorghum mutant lines.

	Summary of Mutant Population		
Amino Acids	Mean (%)	Maximum (%)	Minimum (%)
Taurine	1.29	1.81	0.89
Hydroxyproline	0.08	0.20	0.00
Aspartic Acid	6.45	7.57	5.90
Threonine	3.07	3.40	2.87
Serine	3.97	4.36	3.63
Glutamic Acid	21.70	23.37	19.73
Proline	8.07	8.59	7.40
Lanthionine	0.13	0.28	0.00
Glycine	2.88	3.32	2.43
Alanine	9.51	10.22	8.80
Cysteine	1.67	2.07	1.15
Valine	5.03	5.32	4.71
Methionine	1.61	2.16	1.08
Isoleucine	4.22	4.46	3.89
Leucine	13.98	14.94	12.97
Tyrosine	3.51	3.82	2.83
Phenylalanine	5.43	5.72	5.05
Hydroxylysine	0.19	0.37	0.08
Ornithine	0.05	0.09	0.00
Lysine	2.08	2.66	1.78
Histidine	2.22	2.45	1.92
Arginine	3.38	4.28	2.87
Tryptophan	0.82	0.98	0.67

## Data Availability

Not applicable.

## Supplementary Materials

The following supporting information can be downloaded at: https://www.mdpi.com/article/10.3390/plants12081662/s1, Figure S1: Relationship between amino acids concertation (g/100 g of seeds) and protein concentration (g/100 g of seeds) in seed of mutant lines; Figure S2: Number of mutant lines with various concentrations of non- protein amino acids such as taurine, lanthionine, and ornithine etc.

## References

[1] Bakari H., Ruben Z.F., Roger D.D., Cedric D., Guillaume P., Pascal D., Philippe M., Gwendoline C. (2022). Sorghum (*Sorghum bicolor* L. Moench) and Its Main Parts (By-Products) as Promising Sustainable Sources of Value-Added Ingredients. Waste Biomass Valoriz..

[2] Espitia-Hernández P., Chávez González M., Ascacio-Valdés J., Dávila-Medina D., Flores-Naveda A., Silva T., Ruelas-Chacón X., Sepúlveda-Torre L. (2022). Sorghum (*Sorghum bicolor* L. Moench): Chemical Composition and Its Health Benefits. https://www.researchgate.net/publication/364347835_Sorghum_Sorghum_bicolor_L_Moench_chemical_composition_and_its_health_benefits.

[3] Wong J.H., Lau T., Cai N., Singh J., Pedersen J.F., Vensel W.H., Hurkman W.J., Wilson J.D., Lemaux P.G., Buchanan B.B. (2009). Digestibility of protein and starch from sorghum (*Sorghum bicolor*) is linked to biochemical and structural features of grain endosperm. J. Cereal Sci..

[4] López Ortiz N.C., Tique M.M., Pérez Lavalle L.d.S. (2011). Contribution to the research of sorghum (*Sorghum bicolor* (L.) Moench) for human nutrition. Perspect. Nutr. Hum..

[5] Ebadi M., Sedghi M., Golian A., Ahmadi H. (2011). Prediction of the true digestible amino acid contents from the chemical composition of sorghum grain for poultry. Poult. Sci..

[6] Getachew G., Putnam D.H., De Ben C.M., De Peters E.J. (2016). Potential of sorghum as an alternative to corn forage. Am. J. Plant Sci..

[7] Sm I., Bean S., Loerger B., Hayes C., Emendack Y., Svk J. (2023). Comparative assessment of grain quality in tannin versus non-tannin sorghums in the sorghum association panel. Cereal Chem..

[8] Lin H., Bean S., Tilley M., Peiris K., Brabec D. (2021). Qualitative and quantitative analysis of sorghum grain composition including protein and tannins using ATR-FTIR spectroscopy. Food Anal. Methods.

[9] Zarei M., Amirkolaei A.K., Trushenski J.T., Sealey W.M., Schwarz M.H., Ovissipour R. (2022). Sorghum as a Potential Valuable Aquafeed Ingredient: Nutritional Quality and Digestibility. Agriculture.

[10] Widowati S., Luna P. (2022). Nutritional and functional properties of sorghum (*Sorghum bicolor* (L.) Moench)-based products and potential valorisation of Sorghum Bran. IOP Conference Series: Earth and Environmental Science.

[11] Espinosa-Ramírez J., Serna-Saldívar S.O. (2016). Functionality and characterization of kafirin-rich protein extracts from different whole and decorticated sorghum genotypes. J. Cereal Sci..

[12] Afoakwah N.A., Mahunu G.K., Mariod A.A. (2022). The Quality Aspect and Safety of Some Traditional Fermented Product from Sorghum and millet. African Fermented Food Products-New Trends.

[13] Abah C., Ishiwu C., Obiegbuna J., Oladejo A. (2020). Sorghum grains: Nutritional composition, functional properties and its food applications. Eur. J. Nutr. Food Saf..

[14] Belton P., Delgadillo I., Halford N., Shewry P. (2006). Kafirin structure and functionality. J. Cereal Sci..

[15] Bean S., Wilson J., Moreau R., Galant A., Awika J., Kaufman R., Adrianos S., Ioerger B. (2019). Structure and composition of the sorghum grain. Sorghum: A State Art Future Perspetives.

[16] Taylor J.R., Duodu K.G. (2022). Resistant-Type Starch in Sorghum Foods—Factors Involved and Health Implications. Starch-Stärke.

[17] Xu X., Bean S., Wu X., Shi Y.-C. (2022). Effects of protein digestion on in vitro digestibility of starch in sorghum differing in endosperm hardness and flour particle size. Food Chem..

[18] Shewry P.R. (2007). Improving the protein content and composition of cereal grain. J. Cereal Sci..

[19] Li C., Song R. (2020). The regulation of zein biosynthesis in maize endosperm. Theor. Appl. Genet..

[20] Duodu K., Nunes A., Delgadillo I., Parker M., Mills E., Belton P., Taylor J. (2002). Effect of grain structure and cooking on sorghum and maize in vitro protein digestibility. J. Cereal Sci..

[21] Benmoussa M., Chandrashekar A., Ejeta G., Hamaker B.R. (2015). Cellular response to the high protein digestibility/high-lysine (hdhl) sorghum mutation. Plant Sci..

[22] Da Silva L.S., Taylor J., Taylor J.R. (2011). Transgenic sorghum with altered kafirin synthesis: Kafirin solubility, polymerization, and protein digestion. J. Agric. Food Chem..

[23] Abdualrahman M.A.Y., Ma H., Yagoub A.E.A., Zhou C., Ali A.O., Yang W. (2019). Nutritional value, protein quality and antioxidant activity of Sudanese sorghum-based kissra bread fortified with bambara groundnut (*Voandzeia subterranea*) seed flour. J. Saudi Soc. Agric. Sci..

[24] Wu Y., Yuan L., Guo X., Holding D.R., Messing J. (2013). Mutation in the seed storage protein kafirin creates a high-value food trait in sorghum. Nat. Commun..

[25] Taylor J., Taylor J.R. (2018). Making kafirin, the sorghum prolamin, into a viable alternative protein source. J. Am. Oil Chem. Soc..

[26] Fatima A., Srivastava S. (2017). Role of essential amino acids in human body and its presence in spirulina. Int. J. Appl. Home Sci..

[27] Heger J. (2003). Essential to non-essential amino acid ratios. Amino Acids in Animal Nutrition.

[28] Takahashi T., Toda E., B Singh R., De Meester F., Wilczynska A., Wilson D., Juneja L.R. (2011). Essential and non-essential amino acids in relation to glutamate. Open Nutraceuticals J..

[29] Elkonin L., Panin V., Gerashchenkov G., Kenzhegulov O. (2019). Improvement of sorghum grain quality using modern genetic tools. Current Challenges in Plant Genetics, Genomics, Bioinformatics, and Biotechnology, Proceedings of the 5th International Scientific Conference (PlantGen2019), Novosibirsk, Russia, 24–29 June 2019.

[30] Chakraborty S., Chakraborty N., Agrawal L., Ghosh S., Narula K., Shekhar S., Naik P.S., Pande P., Chakrborti S.K., Datta A. (2010). Next-generation protein-rich potato expressing the seed protein gene AmA1 is a result of proteome rebalancing in transgenic tuber. Proc. Natl. Acad. Sci. USA.

[31] Jiang S.-Y., Ma A., Xie L., Ramachandran S. (2016). Improving protein content and quality by over-expressing artificially synthetic fusion proteins with high lysine and threonine constituent in rice plants. Sci. Rep..

[32] Newell-McGloughlin M. (2008). Nutritionally improved agricultural crops. Plant Physiol..

[33] Zhao Z.-y., Glassman K., Sewalt V., Wang N., Miller M., Chang S., Thompson T., Catron S., Wu E., Bidney D. (2003). Nutritionally improved transgenic sorghum. Plant Biotechnology 2002 and Beyond.

[34] Richardson A.E., Hake S. (2022). The power of classic maize mutants: Driving forward our fundamental understanding of plants. Plant Cell.

[35] Zenda T., Liu S., Dong A., Duan H. (2021). Advances in cereal crop genomics for resilience under climate change. Life.

[36] Deng M., Wang Y., Kuzma M., Chalifoux M., Tremblay L., Yang S., Ying J., Sample A., Wang H.M., Griffiths R. (2020). Activation tagging identifies Arabidopsis transcription factor AtMYB68 for heat and drought tolerance at yield determining reproductive stages. Plant J..

[37] Li Z., Jiang L., Ma Y., Wei Z., Hong H., Liu Z., Lei J., Liu Y., Guan R., Guo Y. (2017). Development and utilization of a new chemically-induced soybean library with a high mutation density. J. Integr. Plant Biol..

[38] McCallum C.M., Comai L., Greene E.A., Henikoff S. (2000). Targeting Induced Local Lesions IN Genomes (TILLING) for plant functional genomics. Plant Physiol..

[39] Martín B., Ramiro M., Martínez-Zapater J.M., Alonso-Blanco C. (2009). A high-density collection of EMS-induced mutations for TILLING in Landsberg erecta genetic background of Arabidopsis. BMC Plant Biol..

[40] Greene E.A., Codomo C.A., Taylor N.E., Henikoff J.G., Till B.J., Reynolds S.H., Enns L.C., Burtner C., Johnson J.E., Odden A.R. (2003). Spectrum of chemically induced mutations from a large-scale reverse-genetic screen in Arabidopsis. Genetics.

[41] Wu J.-L., Wu C., Lei C., Baraoidan M., Bordeos A., Madamba M.R.S., Ramos-Pamplona M., Mauleon R., Portugal A., Ulat V.J. (2005). Chemical-and irradiation-induced mutants of indica rice IR64 for forward and reverse genetics. Plant Mol. Biol..

[42] Mohapatra T., Robin S., Sarla N., Sheshashayee M., Singh A., Singh K., Singh N., Amitha Mithra S., Sharma R. (2014). EMS induced mutants of upland rice variety Nagina22: Generation and characterization. Proc. Indian Natl. Sci. Acad..

[43] Slade A.J., Fuerstenberg S.I., Loeffler D., Steine M.N., Facciotti D. (2005). A reverse genetic, nontransgenic approach to wheat crop improvement by TILLING. Nat. Biotechnol..

[44] Rawat N., Sehgal S.K., Joshi A., Rothe N., Wilson D.L., McGraw N., Vadlani P.V., Li W., Gill B.S. (2012). A diploid wheat TILLING resource for wheat functional genomics. BMC Plant Biol..

[45] Weil C.F., Monde R.A. (2007). Getting the point—Mutations in maize. Crop Sci..

[46] Lu X., Liu J., Ren W., Yang Q., Chai Z., Chen R., Wang L., Zhao J., Lang Z., Wang H. (2018). Gene-indexed mutations in maize. Mol. Plant.

[47] Caldwell D.G., McCallum N., Shaw P., Muehlbauer G.J., Marshall D.F., Waugh R. (2004). A structured mutant population for forward and reverse genetics in Barley (*Hordeum vulgare* L.). Plant J..

[48] Xin Z., Li Wang M., Barkley N.A., Burow G., Franks C., Pederson G., Burke J. (2008). Applying genotyping (TILLING) and phenotyping analyses to elucidate gene function in a chemically induced sorghum mutant population. BMC Plant Biol..

[49] Jiao Y., Burke J., Chopra R., Burow G., Chen J., Wang B., Hayes C., Emendack Y., Ware D., Xin Z. (2016). A sorghum mutant resource as an efficient platform for gene discovery in grasses. Plant Cell.

[50] Mba C. (2013). Induced mutations unleash the potentials of plant genetic resources for food and agriculture. Agronomy.

[51] Rakow G. (1973). Selektion auf Linol-und Linolensauregehalt in Rapssamen nach mutagener Behandlung. Z Pflanzenzucht.

[52] Wang N., Wang Y., Tian F., King G.J., Zhang C., Long Y., Shi L., Meng J. (2008). A functional genomics resource for *Brassica napus*: Development of an EMS mutagenized population and discovery of FAE1 point mutations by TILLING. New Phytol..

[53] Dierking E.C., Bilyeu K.D. (2009). New sources of soybean seed meal and oil composition traits identified through TILLING. BMC Plant Biol..

[54] Kahl G., Meksem K. (2008). The Handbook of Plant Functional Genomics: Concepts and Protocols.

[55] Lakhssassi N., Zhou Z., Liu S., Colantonio V., AbuGhazaleh A., Meksem K. (2017). Characterization of the FAD2 gene family in soybean reveals the limitations of gel-based TILLING in genes with high copy number. Front. Plant Sci..

[56] Tang S., Liu D.X., Lu S., Yu L., Li Y., Lin S., Li L., Du Z., Liu X., Li X. (2020). Development and screening of EMS mutants with altered seed oil content or fatty acid composition in *Brassica napus*. Plant J..

[57] Paulis J., Bietz J., Bogyo T., Nelsen T., Darrah L., Zuber M. (1992). Expression of A/B zeins in single and double maize endosperm mutants. Theor. Appl. Genet..

[58] Zhou Z., Song L., Zhang X., Li X., Yan N., Xia R., Zhu H., Weng J., Hao Z., Zhang D. (2016). Introgression of opaque2 into waxy maize causes extensive biochemical and proteomic changes in endosperm. PLoS ONE.

[59] Bello B.K., Hou Y., Zhao J., Jiao G., Wu Y., Li Z., Wang Y., Tong X., Wang W., Yuan W. (2019). NF-YB 1-YC 12-bHLH 144 complex directly activates Wx to regulate grain quality in rice (*Oryza sativa* L.). Plant Biotechnol. J..

[60] Lu C., Xin Z., Ren Z., Miquel M., Browse J. (2009). An enzyme regulating triacylglycerol composition is encoded by the ROD1 gene of Arabidopsis. Proc. Natl. Acad. Sci. USA.

[61] Chen B., Heneen W. (1989). Evidence for spontaneous diploid androgenesis in *Brassica napus* L.. Sex. Plant Reprod..

[62] Emrani N., Harloff H.-J., Gudi O., Kopisch-Obuch F., Jung C. (2015). Reduction in sinapine content in rapeseed (*Brassica napus* L.) by induced mutations in sinapine biosynthesis genes. Mol. Breed..

[63] Hussain S., Khan W.M., Khan M.S., Akhtar N., Umar N., Ali S., Ahmed S., Shah S.S. (2017). Mutagenic effect of sodium azide (NaN3) on M2 generation of *Brassica napus* L.(variety Dunkled). Pure Appl. Biol..

[64] Kumar P., Hossain F., Singh N., Choudhary P., Gupta M., Singh V., Chikappa G., Kumar R., Kumar B., Jat S. (2019). Nutritional quality improvement in maize (*Zea mays*): Progress and challenges. Indian J. Agric. Sci..

[65] Tulu D. (2022). Breeding Maize (*Zea mays* L.) to Improve Protein Quality in the Endosperm: A Review. Adv. Life Sci. Technol..

[66] Simons J.M., Herbert T.C., Kauffman C., Batete M.Y., Simpson A.T., Katsuki Y., Le D., Amundson D., Buescher E.M., Weil C. (2022). Systematic prediction of EMS-induced mutations in a sorghum mutant population. Plant Direct.

[67] Sattler S.E., Saballos A., Xin Z., Funnell-Harris D.L., Vermerris W., Pedersen J.F. (2014). Characterization of novel sorghum brown midrib mutants from an EMS-mutagenized population. G3 Genes Genomes Genet..

[68] Xin Z., Wang M., Cuevas H.E., Chen J., Harrison M., Pugh N.A., Morris G. (2021). Sorghum genetic, genomic, and breeding resources. Planta.

[69] Wang J., Hu Z., Upadhyaya H.D., Morris G.P. (2020). Genomic signatures of seed mass adaptation to global precipitation gradients in sorghum. Heredity.

[70] Jiao Y., Lee Y.K., Gladman N., Chopra R., Christensen S.A., Regulski M., Burow G., Hayes C., Burke J., Ware D. (2018). MSD1 regulates pedicellate spikelet fertility in sorghum through the jasmonic acid pathway. Nat. Commun..

[71] Talebi A.B., Talebi A.B., Shahrokhifar B. (2012). Ethyl methane sulphonate (EMS) induced mutagenesis in Malaysian rice (cv. MR219) for lethal dose determination. Am. J. Plant Sci..

[72] Chen Y.L., Liang H.L., Ma X.L., Lou S.L., Xie Y.Y., Liu Z.L., Chen L.T., Liu Y.G. (2013). An Efficient Rice Mutagenesis System Based on Suspension-Cultured Cells. J. Integr. Plant Biol..

[73] Font R., del Río-Celestino M., de Haro-Bailón A. (2006). The use of near-infrared spectroscopy (NIRS) in the study of seed quality components in plant breeding programs. Ind. Crops Prod..

[74] Lee H., Cho B.-K., Kim M.S., Lee W.-H., Tewari J., Bae H., Sohn S.-I., Chi H.-Y. (2013). Prediction of crude protein and oil content of soybeans using Raman spectroscopy. Sens. Actuators B Chem..

[75] Zurak D., Kljak K., Grbeša D. (2020). The composition of floury and vitreous endosperm affects starch digestibility kinetics of the whole maize kernel. J. Cereal Sci..

[76] de Borja Reis A.F., Tamagno S., Moro Rosso L.H., Ortez O.A., Naeve S., Ciampitti I.A. (2020). Historical trend on seed amino acid concentration does not follow protein changes in soybeans. Sci. Rep..

[77] Lasztity B. (1996). Dynamics of nutrient accumulation in aboveground parts of sorghum. Novenytermeles.

[78] De Mesa-Stonestreet N.J., Alavi S., Bean S.R. (2010). Sorghum proteins: The concentration, isolation, modification, and food applications of kafirins. J. Food Sci..

[79] Espina M.J., Ahmed C.S., Bernardini A., Adeleke E., Yadegari Z., Arelli P., Pantalone V., Taheri A. (2018). Development and phenotypic screening of an ethyl methane sulfonate mutant population in soybean. Front. Plant Sci..

[80] AOAC (2000). Official Methods of Analysis.

[81] AOAC (2006). Official Methods of Analysis.

[82] Peiris K.H., Bean S.R., Chiluwal A., Perumal R., Jagadish S.K. (2019). Moisture effects on robustness of sorghum grain protein near-infrared spectroscopy calibration. Cereal Chem..

[83] Arthur F., Bean S., Smolensky D., Cox S., Lin H., Peiris K., Peterson J. (2020). Development of *Rhyzopertha dominica* (Coleoptera: Bostrychidae) on sorghum: Quality characteristics and varietal susceptibility. J. Stored Prod. Res..

