# Supplementary material for: Variations in Total Protein and Amino Acids in the Sequenced Sorghum Mutant Library
Source: Plants (Basel). 2023 Apr 15;12(8):1662. doi: 10.3390/plants12081662 (PMC10142022; doi:10.3390/plants12081662)
Supplement: Supplementary file 1 [file plants-12-01662-s001.zip › plants-2309114-supplementary.pdf]

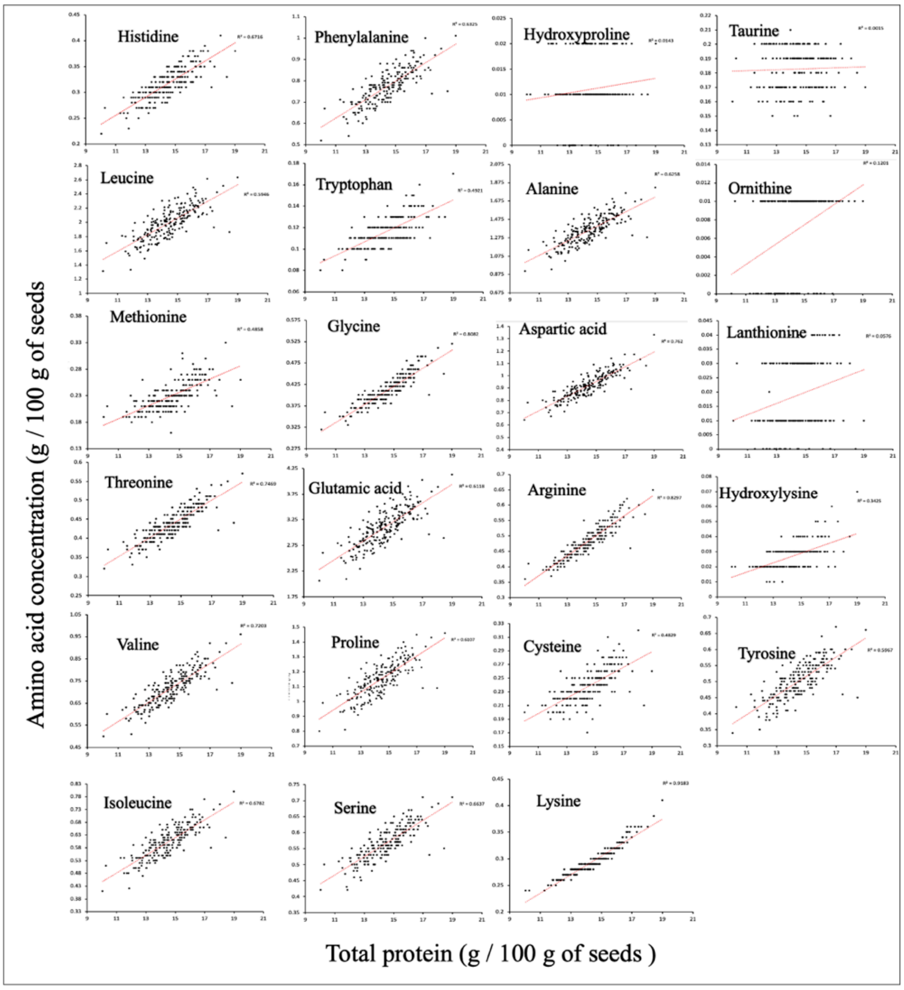

Figure S1. Relationship between amino acids concertation (g/100 g of seeds) and protein concentration (g / 100 g of seeds) in seed of mutant lines. Each point indicates the amino acid concentration in each mutant line. The r value represents the Pearson correlation.

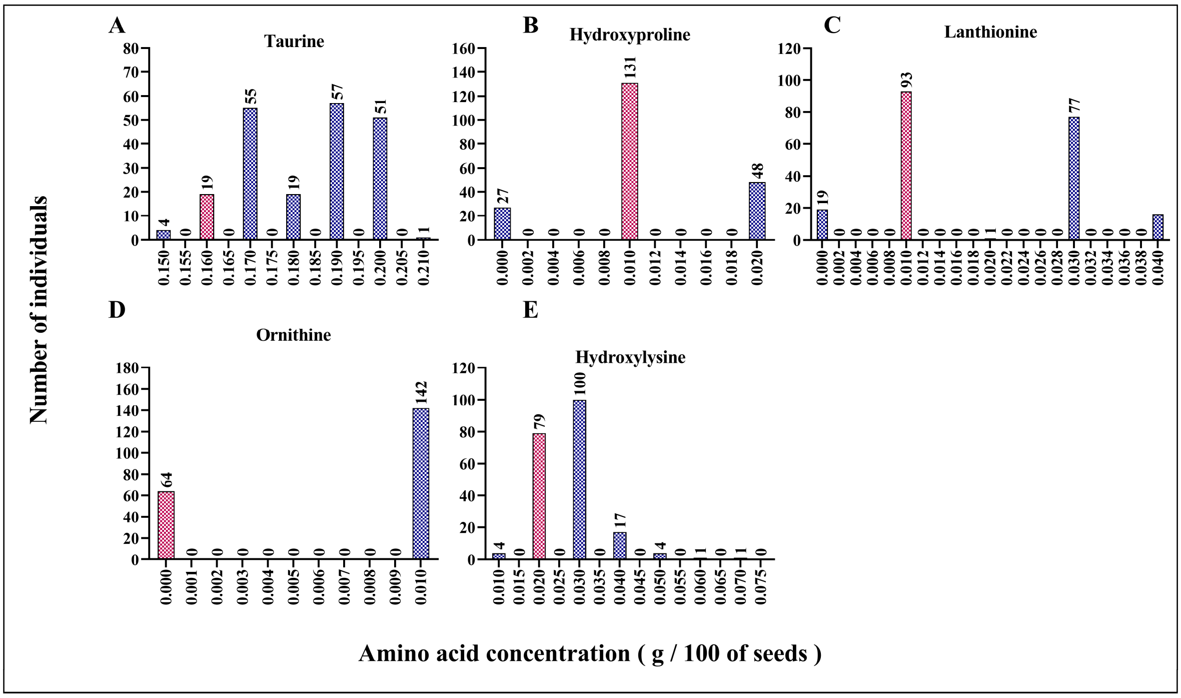

Figure S2. Number of mutant lines with various concentrations of non- protein amino acids such as taurine, lanthionine, and ornithine etc. The x value indicates g/100 gram of dry seed sample. Total protein was extracted from 100 g of seeds and the amino acids were quantified. Control line (BTx623) is indicated by a red bar in each panel.
